# Least squares-based biomass conversion and expansion factors best estimate biomass than ratio-based ones: Statistical evidences based on tropical timber species

**DOI:** 10.1016/j.mex.2018.01.005

**Published:** 2018-01-28

**Authors:** Tarquinio Mateus Magalhães, Rosta Simão Mate

**Affiliations:** Departamento de Engenharia Florestal,Universidade Eduardo Mondlane, Campus Universitário, Edifício no. 1, 257 Maputo, Mozambique

**Keywords:** Biomass, Biomass conversion and expansion factors, Miombo, Mopane

## Abstract

Due to its readiness to convert stem volumes (V) into biomass, national and regional aboveground biomass estimates and greenhouse gas reporting are generally based on biomass conversion and expansion factors (BCEFs). BCEF-based biomass (Ŵ) is computed by the following regression through the origin (RTO): Ŵ = BCEF × V. However, the regression slope (BCEF) is not obtained using least squares (LS); it is obtained as the ratio of observed biomass and stem volume. Therefore, the sum of squares of the residuals is not minimum. This may lead to strongly biased biomass estimates. Furthermore, in this case, the biomass is not modelled. In the present study, it was suggested that BCEFs should be obtained using LS through RTO. The objective of this study was to compare LS-based and ratio-based BCEFs with regard to predictive accuracy and ability. A dataset of 75 trees from 4 species was used for the comparisons.

• LS-based BCEFs were associated with higher predictive accuracy and ability than ratio-based ones.
• It was proved that RTO is appropriated for estimating BCEFs, as the intercept α was consistently not significant.
• Ratio-based BCEFs may lead to seriously biased biomass and carbon stocks estimates.
• BCEFs should be estimated using least squares.

## Graphical abstract

**Figure:**
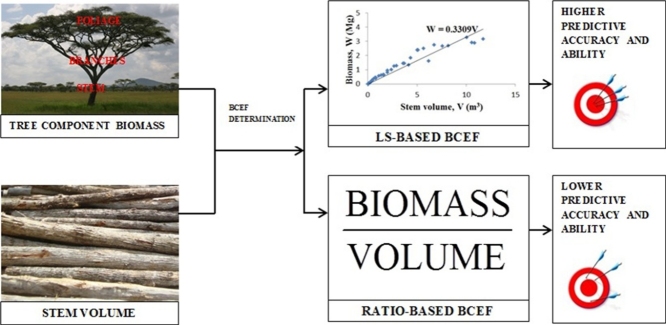

## Methods details

### Background

Forest biomass is a crucial ecological variable for understanding the evolution and potential future changes of the climate system [1]. Therefore, a global assessment of biomass and its dynamics is an essential input to climate change projection models and mitigation and adaptation strategies [1].

Forest biomass can be estimated non-destructively using biomass equations. When biomass equations are fitted using least squares they are called biomass regression equations. Biomass regression equations are developed as linear or non-linear functions of one or more tree-level dimensions. When biomass equations are fitted in such a way that they specify tree component biomass as directly proportional to stem volume, the ratios of proportionality are then called biomass conversion and expansion factors (BCEFs) [2].

National and regional aboveground biomass (AGB) estimates and greenhouse gas (GHG) reporting are generally based on BCEFs [[2], [3]], mainly because of its readiness to convert standing stem volumes from forest inventories into different tree component biomasses [4], including the non-commercial components (foliage, needles, branches, root system, etc.) [5].

BCEF-based biomass is computed by the following equation

(1) Ŵ = BCEF × V

where Ŵ is the predicted tree component biomass and V stem volume.

Eq. (1) is, actually, a regression through the origin (RTO) of biomass on stem volume where, therefore, the BCEF value is the slope. However, the regression slope (BCEF) is not obtained using least squares (LS), but as the ratio of observed tree component biomass and stem volume [[2], [6]]. Hence, the sum of squares of the residuals is not minimum, which may lead to strongly biased biomass estimates. Furthermore, in this case, the biomass is not modelled [2].

The assumption behind Eq. (1) is that tree component biomass is directly proportional to stem volume and that if stem volume is zero, then concurrently, tree component biomass is zero, which is true. Therefore, the ratio estimators are deemed appropriate [[7], [8], [9], [10]], and BCEF is then computed as such (i.e. using ratio estimators). Nonetheless, as mentioned previously, it fails by not using least squares and not modelling the biomass. Therefore, fitting Eq. (1) using RTO − i.e. obtaining BCEF in Eq. (1) using least squares − might provide more accurate biomass estimates than using ratio estimators (ratio-based BCEFs).

The objective of this study was to compare LS-based and ratio-based tree component BCEFs with regard to predictive accuracy and ability. The study addressed the following research question: do LS-based- and ratio-based BCEFs differ in terms of predictive accuracy and ability? It was hypothesized that LS-based tree component BCEFs provide most accurate and reliable estimates.

## Data acquisition

The study was conducted in Mozambique (18° 15′S, 35° 00′E), in Gaza, Inhambane and Sofala provinces. Seventy five (75) trees from four valuable timber species were destructively sampled for biomass and volume estimation, namely: *Colophospermum mopane* Kirk ex J. Leonard, *Afzelia quanzensis* Welw., *Millettia stuhlmannii* Taub., and *Pterocarpus angolensis* DC.

*C. mopane* was harvested in Mabalane district, Gaza province. Other three species (*A. quanzensis, M. stuhlmannii, and P. angolensis*) were harvested in Funhalouro district (Inhambane province) and Cheringoma district (Sofala province).

Seventeen (17), 24, 15, and 19 sample trees of *C. mopane, A. quanzensis, M. stuhlmannii,* and *P. angolensis* (Table 1), respectively, were sampled. *C. mopane* was harvested from Mopane woodlands and the remaining tree species from Miombo woodlands.

**Table 1:** Summary statistics of the data.

Statistic	DBH (cm)	TH (m)	Stem volume (m^3^)	Tree component dry-weights (kg)				
				Stem	Branches	Foliage	Crown	AGB
*C. mopane*								
Min	5.00	4.72	0.01	2.80	1.40	0.50	1.90	4.70
Mean (±SE)	50.03 (±5.33)	15.87 (±0.80)	3.11 (±0.58)	1095.2 (±175.14)	1115.40 (±203.70)	23.50 (±3.27)	1138.90 (±206.38)	2434.12 (±377.75)
Max	109.20	22.60	10.55	3299.10	3808.40	59.30	3865.80	7164.90

*A. quanzensis*								
Min	13.50	10.00	0.37	14.22	57.29	0.66	59.45	106.90
Mean (±SE)	33.80 (±2.58)	14.96 (±0.47)	0.98 (±0.13)	552.00 (±98.54)	296.87 (±37.65)	18.71 (±3.89)	315.59 (±39.41)	867.58 (±112.16)
Max	61.10	19.00	3.16	1555.60	666.92	77.41	701.92	2016.80

*M. stuhlmannii*								
Min	21.00	10.50	0.20	296.48	42.47	0.96	51.27	411.44
Mean (±SE)	34.78 (±2.13)	14.97 (±0.49)	0.84 (±0.14)	782.33 (±88.16)	222.30 (±41.68)	11.17 (±1.92)	233.47 (±41.83)	1015.8 (±113.17)
Max	52.20	17.00	1.93	1411.94	658.82	27.05	673.71	2085.65

*P. angolensis*								
Min	14.00	6.50	0.08	16.15	16.00	1.41	21.00	52.30
Mean (±SE)	26.96 (±2.18)	11.44 (±0.52)	0.35 (±0.04)	162.38 (±30.91)	156.90 (±30.50)	6.96 (±1.06)	163.87 (±30.87)	326.25 (±54.95)
Max	46.50	15.00	0.70	595.80	516.10	17.54	525.40	1121.20

After measuring the diameter at breast height (DBH), the trees were felled considering a predefined stump height of 20 cm. The aboveground portion of the tree was divided into following biomass components: stem, branches, foliage, and crown (branches + foliage).

The stem was divided into 5 segments equal in length and the diameter of each segment was measured at the midpoint. The volume of the stem was determined using Hohenadl’s formula [6]. Each segment was fresh-weighted in the field and a disc sample removed on the top of it for oven-drying and subsequent dry-weighting. Discs were oven-dried at 105 °C until constant mass. The dry mass of each segment was obtained by multiplying the ratio of oven-dry- to fresh mass of the disc by the relevant fresh mass of the segment. The dry mass of the stem was obtained as the sum of the dry masses of the constituent segments.

After removing the leaves, each primary branch (along with its secondary and higher-order branches, and twigs) was fresh-weighted in the field. A sample composed by a disc removed from the primary branch, samples of secondary and higher-order branches and twigs were taken from each primary branch. Dry mass of each primary branch was obtained similarly to that of each stem segment.

All the foliage from the crown was measured in the field and a sample of approximately 5% of the fresh mass collected for oven-drying. The dry mass of the foliage was obtained similarly to that of each stem segment.

## Analyses

Before computing the BCEFs, the Shapiro-Wilk normality test and normal Quantile–Quantile (Q-Q) plots were used to detect departures of each tree component biomass (the response variable) from normality (see Appendices 1 and 2 of Supplementary materials). Shapiro-Wilk normality test and residual Q–Q plots were also used to diagnose the residual distribution (non-normality or normality of the residuals) using ordinary linear regression (see Appendices 3 and 4 of Supplementary materials).

Thus, LS-based BCEFs were obtained using generalized linear model if the response variable (biomass) was found to have a residual distribution other than a normal distribution; and using ordinary linear regression if the residual distribution was normal.

All the residual of all tree components of all species were found to be normally distributed, except the foliage biomass of *A. quanzensis* and *P. angolensis* and AGB of *P. angolensis*.

Ratio-based- and LS-based BCEFs were compared with regard to predictive accuracy and ability.

The predictive accuracy was determined by the following sources of errors in model prediction: (1) error due to model misspecification, (2) error due to uncertainty in the model parameter estimates, and (3) error due to residual variability around model prediction.

Error due to model misspecification is here expressed by Akaike Information Criterion (AIC) [11], as it is a measure of a relative quality of statistical models for a given set of data. The error due to uncertainty in the model parameter estimates is expressed by the standard errors of the regression parameters [12]: standard error of the BCEFs, in this case. In turn, the error due to residual variability around model prediction is here expressed by coefficient of variation of the residuals (CVr) and Furnivaĺs index of fit (FI) [[2], [12]].

The predictive ability is expressed by the mean quadratic error of prediction (MEP) [[13], [14]], and model prediction error (MPE) [15]. MEP is defined by Eq. (2) [[13], [14]].

(2) $$MEP=\frac{1}{n}\sum_{i=1}^{n}\frac{e_{i}^{2}}{{\left(1-H_{ii}\right)}^{2}}$$

where e_i_^2^ is the square of model residual and H_ii_ is the diagonal element of the projection matrix H.

MPE was estimated by K–fold cross-validation (K = 10) using cvFit function from the package “cvTools” [15] of R software [16].

The lower the MEP and MPE, the better the models in terms of predictive ability.

## Comparative results: ratio-based- vs. LS-based BCEFs

### Predictive accuracy

**Fig. 1:**
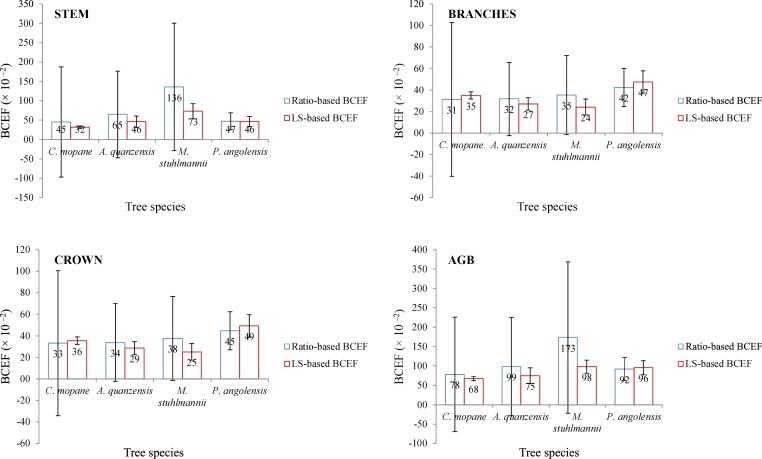
Significance of tree component BCEFs. The error bars indicate 95% confidence interval (CI) computed as CI = t × SE; where t is the critical value of t distribution at 95% of probability and n − 2 degrees of freedom; n is the sample size; and SE is the standard error.

The errors due to model misspecification of LS-based BCEFs, as judged by AIC, were up to 115% smaller than those of ratio-based ones (Table 2, Table 3). The standard errors of the parameters (BCEFs − slopes) varied from 8 to 333% for ratio-based BCEFs and from 4 to 15% for LS-based BCEFs. The errors due to uncertainty in the model parameter estimates of LS-based BCEFs were up to 97% smaller when compared to those of ratio-based ones. Thus, ratio-based BCEFs were associated with wider confidence intervals (Fig. 1); and for all tree components and species, except for *P. angolensis,* ratio-based BCEFs were found not to be statistically significant (Fig. 1).

**Table 2:** Ratio-based- and LS-based BCEFs for *C. mopane* and *A. quanzensis.*

Biomass component	BCEF [Mg m ^−3^]	AIC	SE [%]	CVr [%]	FI	MEP	MPE
Ratio-based BCEF (*C. mopane*)							
Stem	0.4517	36.4	147.5	62.7	0.4462	0.4436	1.3033
Branches	0.3124	13.1	107.5	31.0	0.1514	0.1128	1.3817
Foliage	0.0197	−42.4	333.0	286.3	0.0009	0.0043	0.0274
Crown	0.3320	32.1	95.2	28.6	0.1670	0.2350	1.4062
AGB	0.7837	37.7	88.3	31.9	1.0173	0.4787	2.6892

LS-based BCEF (*C. mopane*)							
Stem	0.3206	9.96	4.9	27.1	0.1318	0.1054	0.2743
Branches	0.3498	10.1	4.5	26.7	0.0842	0.1252	0.2569
Foliage	0.0057	−91.2	13.9	64.5	0.0002	0.0003	0.0142
Crown	0.3556	11	4.6	26.9	0.0943	0.1326	0.2629
AGB	0.6762	27.7	3.9	22.4	0.3930	0.3474	0.4346

Ratio-based BCEF (*A. quanzensis*)							
Stem	0.6487	40.5	83.1	80.9	0.1952	0.2907	0.6347
Branches	0.3174	−16.7	51.6	45.7	0.0523	0.0269	0.3080
Foliage	0.0205	−118.2	96.4	87.6	0.0007	0.0009	0.0190
Crown	0.3379	−13.8	51.6	45.7	0.0584	0.0363	0.3251
AGB	0.9866	46.5	62.0	58.4	0.3820	0.4745	0.9221

LS-based BCEF (*A. quanzensis*)							
Stem	0.4616	38.3	15.3	64.1	0.1789	0.1866	0.4485
Branches	0.2697	−17.6	10.3	33.1	0.0492	0.0174	0.1461
Foliage	0.0159	−118.0	14.7	74.3	0.0004	0.0004	0.0145
Crown	0.2856	−14.9	10.3	32.9	0.0548	0.0310	0.1533
AGB	0.7472	43.1	13.2	42.1	0.3408	0.3688	0.4770

**Table 3:** Ratio-based and LS-based BCEFs for *M. stuhlmannii* and *P. angolensis.*

Biomass component	BCEF [Mg m ^−3^]	AIC	SE [%]	CVr [%]	FI	MEP	MPE
Ratio-based BCEF (*M. stuhlmannii*)							
Stem	1.3581	36.4	56.1	58.7	0.4096	0.5802	0.7937
Branches	0.3532	−8.6	48.1	269.8	0.3268	0.0288	0.2191
Foliage	0.0234	−78.5	70.8	2092.9	0.0368	0.0003	0.0117
Crown	0.3765	−6.7	48.0	55.1	0.0719	0.0327	0.2285
AGB	1.7346	41.6	52.1	40.3	0.4235	0.8180	0.9934

LS-based BCEF (*M. stuhlmannii*)							
Stem	0.7319	22.1	12.3	37.1	0.2507	0.2169	0.4162
Branches	0.2415	−14.9	14.4	38.0	0.0408	0.0208	0.1339
Foliage	0.0096	−93.8	13.1	54.6	0.0008	0.0000	0.0093
Crown	0.2511	−14.3	14.1	36.8	0.0429	0.0213	0.1349
AGB	0.9830	26.6	8.0	33.2	0.3265	0.2957	0.4662

Ratio-based BCEF (*P. angolensis*)							
Stem	0.4715	−30.6	21.8	46.6	0.0292	0.0185	0.1621
Branches	0.4231	−38.1	19.9	40.4	0.0292	0.0071	0.1699
Foliage	0.0240	−144.3	21.4	55.8	0.0003	0.0002	0.0074
Crown	0.4471	−38.3	18.7	38.4	0.0199	0.0070	0.1764
AGB	0.9186	−18.0	15.5	32.8	0.0585	0.0204	0.3160

LS-based BCEF (*P. angolensis*)							
Stem	0.4634	−38.6	13.3	36.5	0.0242	0.0132	0.0922
Branches	0.4748	−37.2	10.3	31.4	0.0182	0.0042	0.0745
Foliage	0.0177	−147.3	15.3	42.8	0.0002	0.0000	0.0036
Crown	0.4925	−9.2	10.0	33.8	0.0160	0.0052	0.0750
AGB	0.9558	−26.2	8.9	29.1	0.0505	0.0168	0.1101

FI and CVr were also considerably smaller for LS-based biomass models (LS-based BCEFs), denoting thus, smaller error due to residual variability around model prediction for LS-based biomass models when compared to ratio-based biomass models (ratio-based BCEFs).

The three sources of errors in model prediction prove that biomass estimates obtained from LS-based BCEFs are more accurate than those obtained from ratio-based BCEFs; e.g. LS-based BCEFs were associated with higher predictive accuracy than ratio-based BCEFs.

Note that, on average, ratio-based BCEFs were larger than LS-based BCEFs; i.e. LS-based BCEFs indicates lower dry weight per unit of stem volume than ratio-based BCEFs. For example, the ratio-based BCEF for stem and AGB for *M. stuhlmannii* (Table 3) indicate that stem biomass and AGB (in Mg) are 1.36- and 1.73-fold larger than stem volume (in m^3^), respectively; whilst LS-based BCEFs indicate that stem biomass and AGB (in Mg) are 0.73- and 0.98-fold larger than stem volume (in m^3^), respectively.

### Predictive ability

The mean quadratic errors of prediction (MEP) of LS-based BCEFs were up to 100% (range: 15–100%) smaller than those of ratio-based ones (Table 2, Table 3). On the other hand, the model prediction errors (MPE) of LS-based BCEFs were up to 84% (range: 21–84%) smaller than those of ratio-based ones. Thus, the predictive ability was higher for LS-based BCEFs than for ratio-based BCEFs.

## Is RTO appropriated for estimating BCEFs?

In this paper, the *a priori* reason why the regression was forced to pass through the origin is that if stem volume is zero, then concurrently, tree component biomass is zero. However, this fact is not enough to justify the use of RTO since, as argued by Wooldridge [17], “*one serious drawback of RTO is that, if the intercept is different from zero, then the LS estimators of the slope will be severely biased*”. Therefore, it was tested whether the hypothesis of the intercept being equal to zero (α = 0) is data admissible (Table 4).

**Table 4:** Test of hypothesis that the intercept of the regression W = α + βV + ε is equal to zero.

Biomass component	Tree species			
	*C. mopane*	*A. quanzensis*	*M. stuhlmannii*	*P. angolensis*
Stem	W = 0.1835 + 0.2934 V + ε; P-value for α = 0.0584.	W = 0.3570 + 0.3198 V + ε; P-value for α = 0.0666.	W = 0.0059 + 0.7027 V + ε; P-value for α = 0.0556.	W = 0.0065 + 0.4487 V + ε; P-value for α = 0.9046.
Branches	W = 0.0527 + 0.3420 V + ε; P-value for α = 0.6081.	W = 0.1161 + 0.1842 V + ε; P-value for α = 0.0554.	W = 0.0715 + 0.1806 V + ε; P-value for α = 0.2820.	W = −0.0372 + 0.5588 V + ε; P-value for α = 0.3847.
Foliage	W = 0.0103 + 0.0042 V + ε; P-value for α = 0.0343.	W = 0.0114 + 0.0075 V + ε; P-value for α = 0.1348.	W = 0.0110 + 0.0001 V + ε; P-value for α = 0.0030.	W = 0.0038 + 0.0091 V + ε; P-value for α = 0.0982.
Crown	W = 0.0630 + 0.3463 V + ε; P-value for α = 0.5504.	W = 0.0030 + 0.2728 V + ε; P-value for α = 0.6594.	W = 0.0825 + 0.1807 V + ε; P-value for α = 0.2199.	W = −0.0334 + 0.5679 V + ε; P-value for α = 0.4364.
AGB	W = 0.2465 + 0.6397 V + ε; P-value for α = 0.1409.	W = 0.0465 + 0.7072 V + ε; P-value for α = 0.1719.	W = 0.0065 + 0.9522 V + ε; P-value for α = 0.2718.	W = −0.0269 + 1.0166 V + ε; P-value for α = 0.7204.

W = dry weight (biomass), V = stem volume, α = intercept, β = slope, ε = error term.

The intercepts of all models were found not to be significant (Table 4) at significance level of 5%, except for the foliage of *C. mopane* and *M. stuhlmannii*.

## Brief discussion

Biomass regression equations, using easily measurable tree dimensions as independent variables (DBH and tree height), yield the most accurate estimates [[18], [19], [20], [21]], provided that they are obtained from a large number of trees [[1], [22]]. However, due to their readiness in converting available stem volumes into any component biomass and their close link to standard forest inventory results [4], ratio-based BCEFs are the most used in obtaining national and regional AGB estimates and GHG reporting [[2], [3]]. Nevertheless, as shown here, they have a very crude predictive accuracy and ability, mainly because they are not obtained using least squares, not minimizing the sum of squares of the residuals.

LS-based BCEFs, to a certain extent, combine the advantages of biomass regression equations and ratio-based BCEFs. However, it should be noted that LS-based BCEFs might not provide biomass estimates as accurate as biomass regression equations. This is because BCEF-based biomass is dependent on stem volume which, in turn, is dependent on DBH, stem height and, sometimes, form factor, if the volume is computed based on form factor instead of a volume equation. All these variables have their own sources of errors which are propagated when estimating biomass. When using biomass regression equation, however, the biomass is, most of the time, dependent only on DBH alone or on DBH and tree height, minimizing the sources of errors.

The choice of an appropriate biomass equation (e.g. BCEF) is decisive for reducing uncertainties in forest biomass stock estimates [23], especially in the context of Reducing Emissions from Deforestation and Forest Degradation (REDD+). Besides being least accurate and precise, it can be seen from Fig. 1 that, for each 100 m^3^/ha of stem volume, ratio-based BCEFs estimates up to 75 Mg ha ^−1^ (76%) larger biomass than LS-based BCEFs. In this context, ratio-based BCEFs will lead, on average, to overestimation of emission factors (EFs), forest reference and emission levels (FRELs), and will compromise the reliability of the estimates of carbon stock changes. Consequently, with unreliable FRELs, the country or REDD+ projects contribution in mitigating climate change through forest related actions cannot be properly assessed and, moreover, the contributions will be unreliable as well.

One of the most important drivers of forest-cover change and forest degradation in Mozambique is selective forest logging [24], which is mostly concentrated in *A. quanzensis, M. stuhlmannii* and *P. angolensis* [24], 3 of the 4 species under study in this research. Therefore, these species are responsible of a large part of forest-cover change and forest degradation due to forest logging, thus responsible of carbon emissions from forest degradation caused by logging. This highlights the need of accurately estimating the biomass of these species.

Accurately estimating biomass is a critical step in quantifying carbon emission from deforestation and forest degradation and in reducing uncertainties of those emissions. At local and global level, emissions related to forest degradation are poorly quantified [25]. At local level (Mozambique), accurate estimates of biomass of the species responsible for forest-cover change and forest degradation due to selective harvesting may promote better quantification of emission from forest degradation. The total emission from selective harvesting is the sum of (1) extracted log emissions (ELE), (2) logging damage factor (LDF), and (3) logging infrastructure factor (LIF) [25]. LS-based BCEFs may improve significantly the estimates of the first two factors as described below:

Accurate estimates of stem BCEFs of those species will lead to better estimates of ELE, which, according to Pearson et al. [25], are *“emissions resulting from conversion of the log to wood products and the subsequent emissions from retired wood products”*. Accurate estimates of branches, foliage and crown BCEFs of the concerned species will provide better estimates of LDF, defined as emission resulting from decomposition of all the dead wood produced as a result of felling the tree(s) [25], which include the foliage and the branches.

Overall, when compared to ratio-based BCEFs, LS-based BCEFs are a potential tool for better estimating biomass and carbon stocks, emission factors and FRELs, while reducing their uncertainties. Specifically, at local context, since LS-based were developed for tree species that are most selectively harvested thus top responsible for forest degradation caused by logging, LS-based BCEFs of these species may contribute in (better) estimating the country-specific emissions from forest degradation.

## Conclusions

In this study, ratio-based- and LS-based BCEFs were compared in terms of predictive accuracy and ability. LS-based BCEFs were associated with extremely lower (1) error due to model misspecification, (2) error due to uncertainty in the model parameter estimates, and (3) error due to residual variability around model prediction, when compared to ratio-based ones; leading to higher predictive accuracy. LS-based BCEFs had lower values of (1) mean quadratic error of prediction, and (2) model prediction error; leading to higher predictive ability than ratio-based BCEFs.

## References

[1] GTOS (2009). Assessment of the Status of the Development of the Standards for the Terrestrial Essential Climate Variables.

[2] Magalhães T.M. (2015). Live above- and belowground biomass of a Mozambican evergreen forest: a comparison of estimates based on regression equations and biomass expansion factors. For. Ecosyst..

[3] Petersson H., Holma S., Ståhl G., Algera D., Fridman J., Lehtonen A., Lundström A., Mäkipää R. (2012). Individual tree biomass equations or biomass expansion factors for assessment of carbon stock changes in living biomass −a comparative study. For. Ecol. Manage..

[4] Pajitík J., Konôpka B., Lukac M. (2011). Individual biomass factors for beech, oak and pine in Slovakia: a comparative study in young naturally regenerated stands. Trees.

[5] Fang J.Y., Wang Z.M. (2001). Forest biomass estimation at regional and global levels, with special reference to China’s forest biomass. Ecol. Res..

[6] Magalhães T.M., Seifert T. (2015). Tree component biomass expansion factors and root-to-shoot ratio of Lebombo ironwood: measurement uncertainty. Carbon Balance Manage..

[7] Freese F. (1962). Elementary forest sampling. Agriculture Handbook No. 232.

[8] Freese F. (1984). Statistics for Land Managers.

[9] de Vries P.G. (1986). Sampling Theory for Forest Inventory.

[10] Jayaraman K. (1999). A Statistical Manual for Forestry Research.

[11] Akaike H., Petrov B.N., Csaki F. (1973). Information theory as an extension of the maximum likelihood principle. Second International Symposium on Information Theory.

[12] Magalhães T.M. (2017). Site-specific height-diameter and stem volume equations for Lebombo-ironwood. Ann. For. Res..

[13] Militkỳ J., Meloun M. (1993). Use of the mean quadratic error of prediction for the construction of biased linear models. Anal. Chim. Acta.

[14] Krejza J., Světlík J., Bednář P. (2017). Allometric relationship and biomass expansion factors (BEFs) for above- and below-ground biomass prediction and stem volume estimation for ash (*Fraxinus excelsior* L.) and oak (*Quercus robur* L.). Trees.

[15] Alfonso A. (2015). cvTools: Cross-validation Tools for Regression Models (R Package Version 0.3.2).

[16] R Core Team (2016). A Language and Environment for Statistical Computing.

[17] Wooldridge J.M. (2016). Introductory Econometrics: a Modern Approach.

[18] IPCC,, Penman J., Gytarsky M., Hiraishi T., Krug T., Kruger D., Pipatti R., Buendia L., Miwa K., Ngara T., Tanabe K., Wagner F. (2003). Good Practice Guidance for Land Use, Land-Use Change and Forestry.

[19] Jalkanen A., Mäkipää R., Stahl G., Lehtonen A., Petersson H. (2005). Estimation of the biomass stock of trees in Sweden: comparison of biomass equations and age-dependent biomass expansion factors. Ann. For. Sci.

[20] Antonio N., Tome M., Tome J., Soares P., Fontes L. (2007). Effect of tree, stand and site variables on the allometry of *Eucalyptus globulus* tree biomass. Can. J. For. Res..

[21] Soares P., Tome M. (2012). Biomass expansion factors for Eucalyptus globulus stands in Portugal. For. Syst..

[22] Husch B., Beers T.W., Kershaw J.A. (2003). Forest Mensuration.

[23] Rutishauser E., Nooŕan F., Laumonier Y., Halperin J., Rufíie, Hergoualćh K., Verchot L. (2013). Generic allometric models including height best estimate forest biomass and carbon stocks in Indonesia. For. Ecol. Manage..

[24] Sitoe A., Salomão A., Wertz-Kanounnikoff S. (2012). The context of REDD+ in Mozambique: Drivers, agents and institutions. Occasional Paper 79.

[25] Pearson T.R.H., Brown S., Cesarim F.M. (2014). Carbon emission from tropical forest degradation caused by logging. Environ. Res. Lett..

