# Supplementary material for: Least squares-based biomass conversion and expansion factors best estimate biomass than ratio-based ones: Statistical evidences based on tropical timber species
Source: MethodsX. 2018 Jan 28;5:30–8. doi: 10.1016/j.mex.2018.01.005 (PMC6318216; doi:10.1016/j.mex.2018.01.005)
Supplement: Supplementary file 1 [file mmc1.pdf]

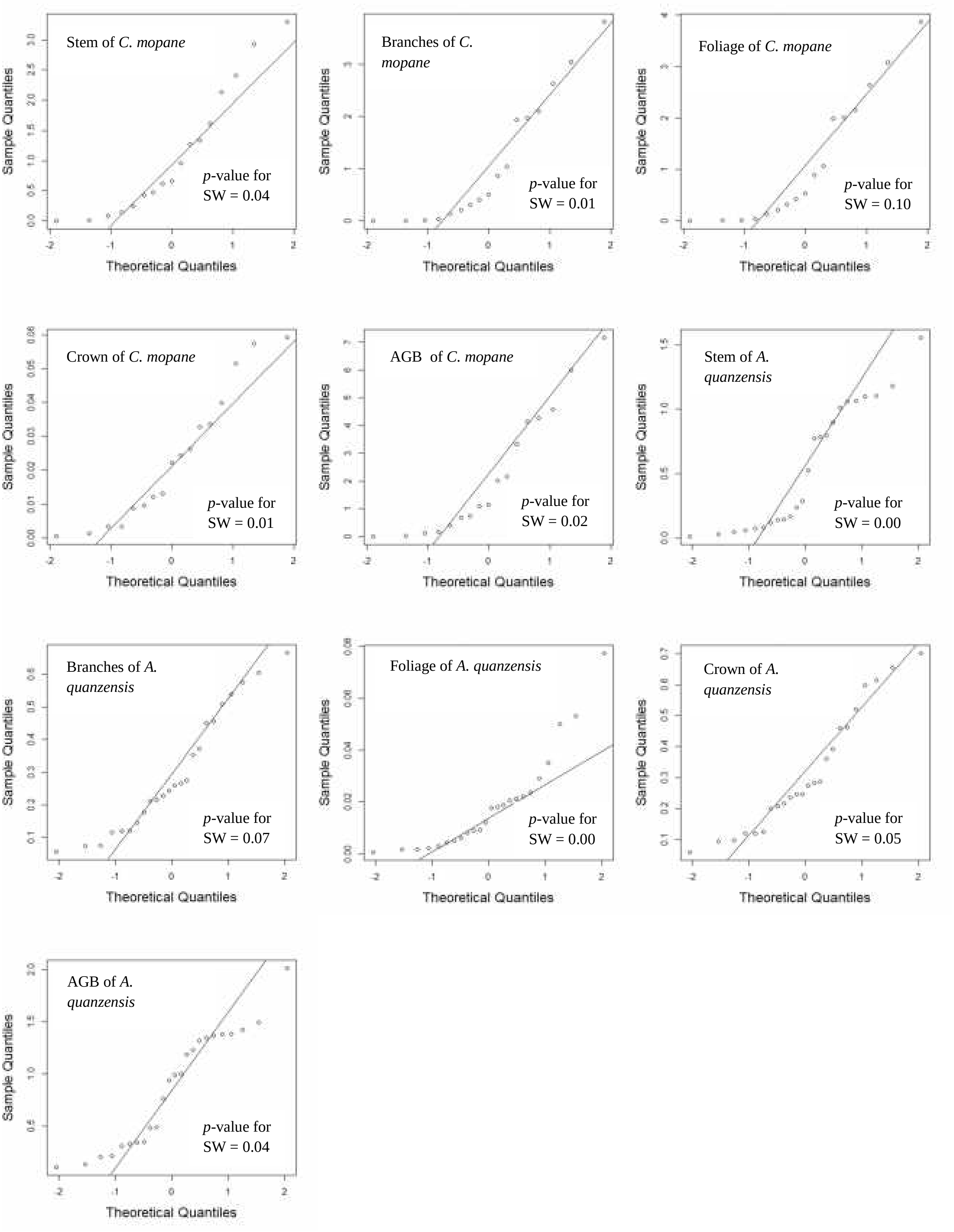

Appendix 1: Quantile-Quantile (Q-Q) plots and Shapiro-Wilk (SW) normality test for tree component biomasses of *C. mopane* and *A. quanzensis*.

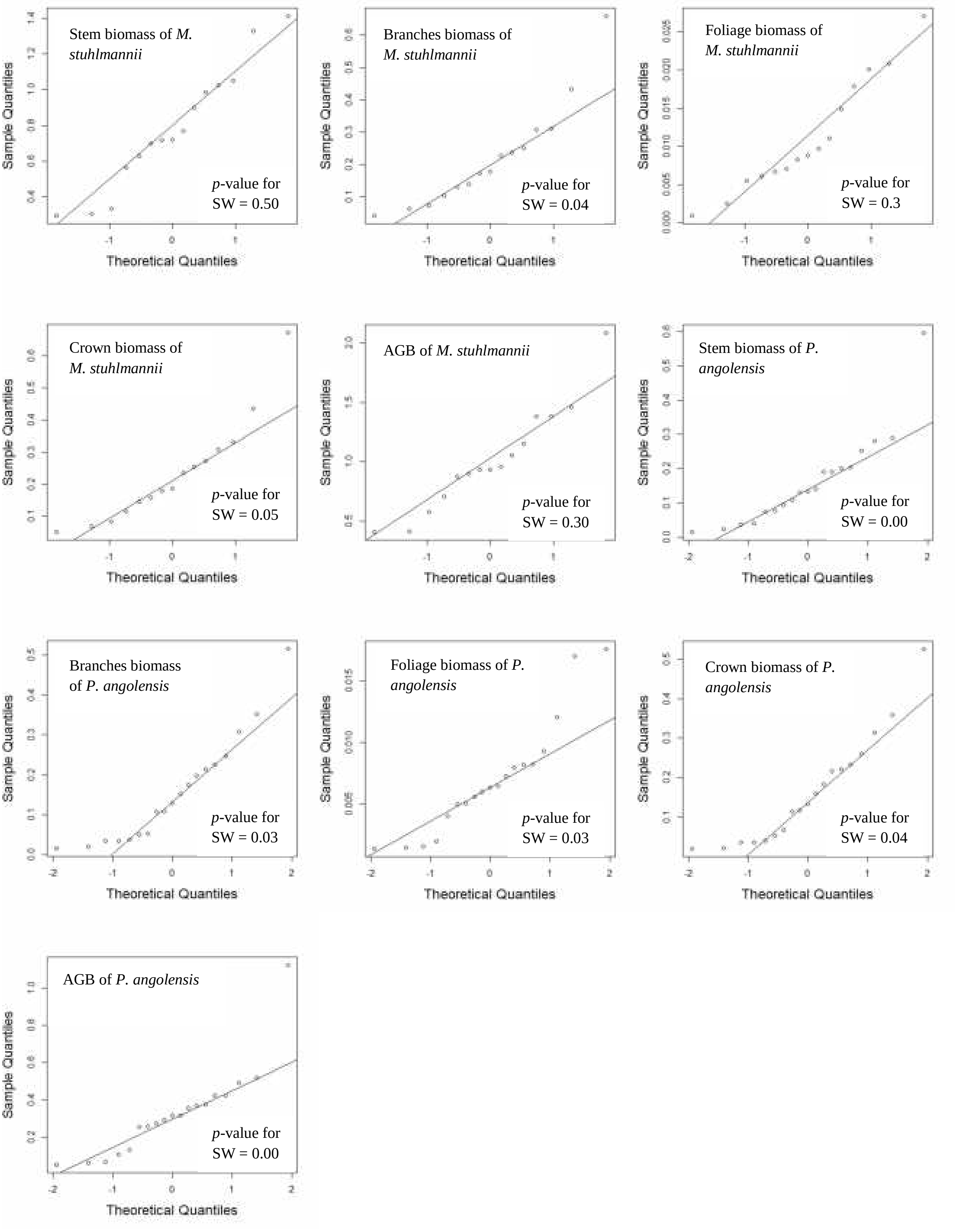

Appendix 2: Quantile-Quantile (Q-Q) plots and Shapiro-Wilk (SW) normality test for tree component biomasses of *M. stuhlmannii* and *P. angolensis.*

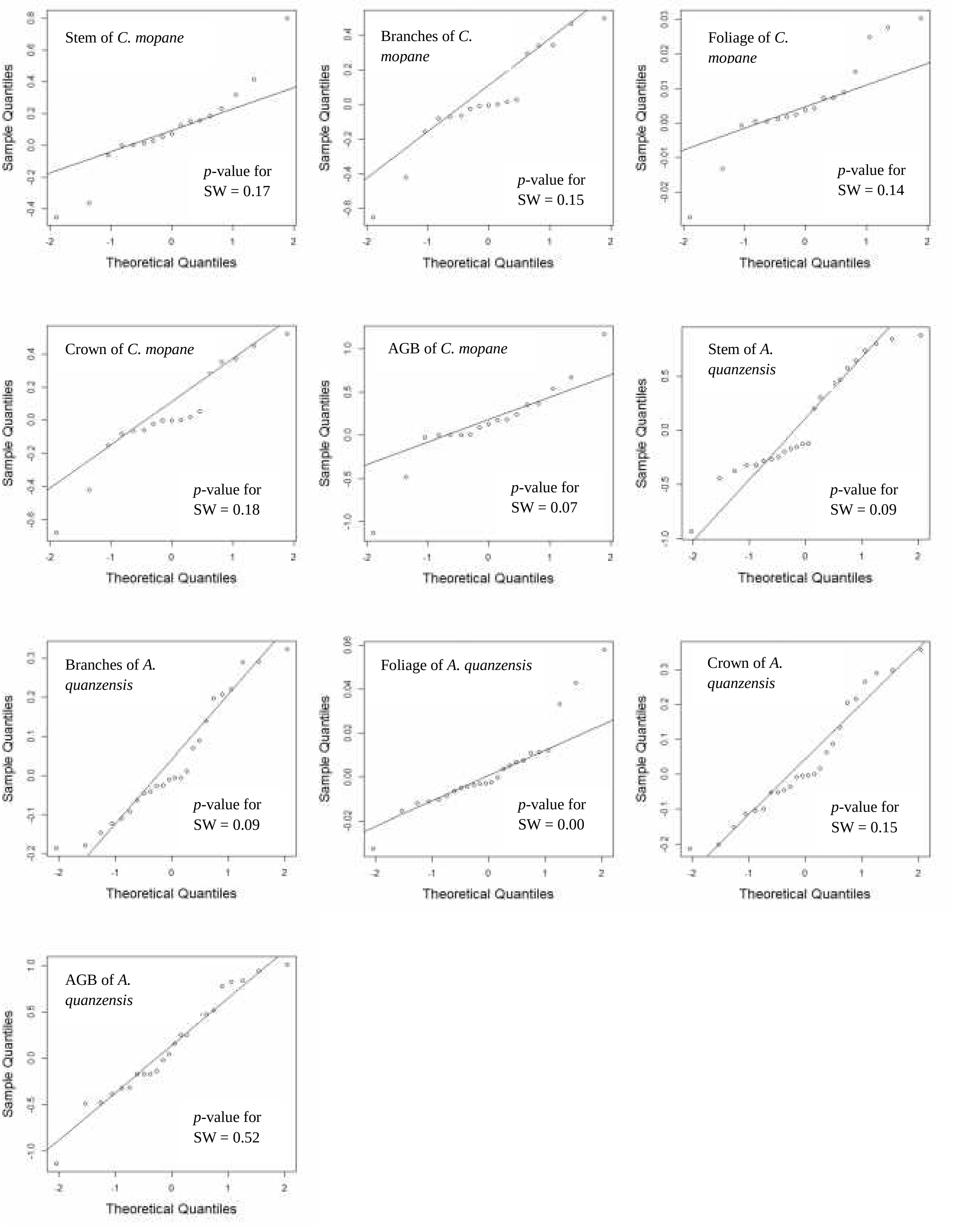

Appendix 3: Residual Quantile-Quantile (Q-Q) plots and Shapiro Wilk (SW) normality test for tree component biomasses of *C. mopane* and *A. quanzensis*.

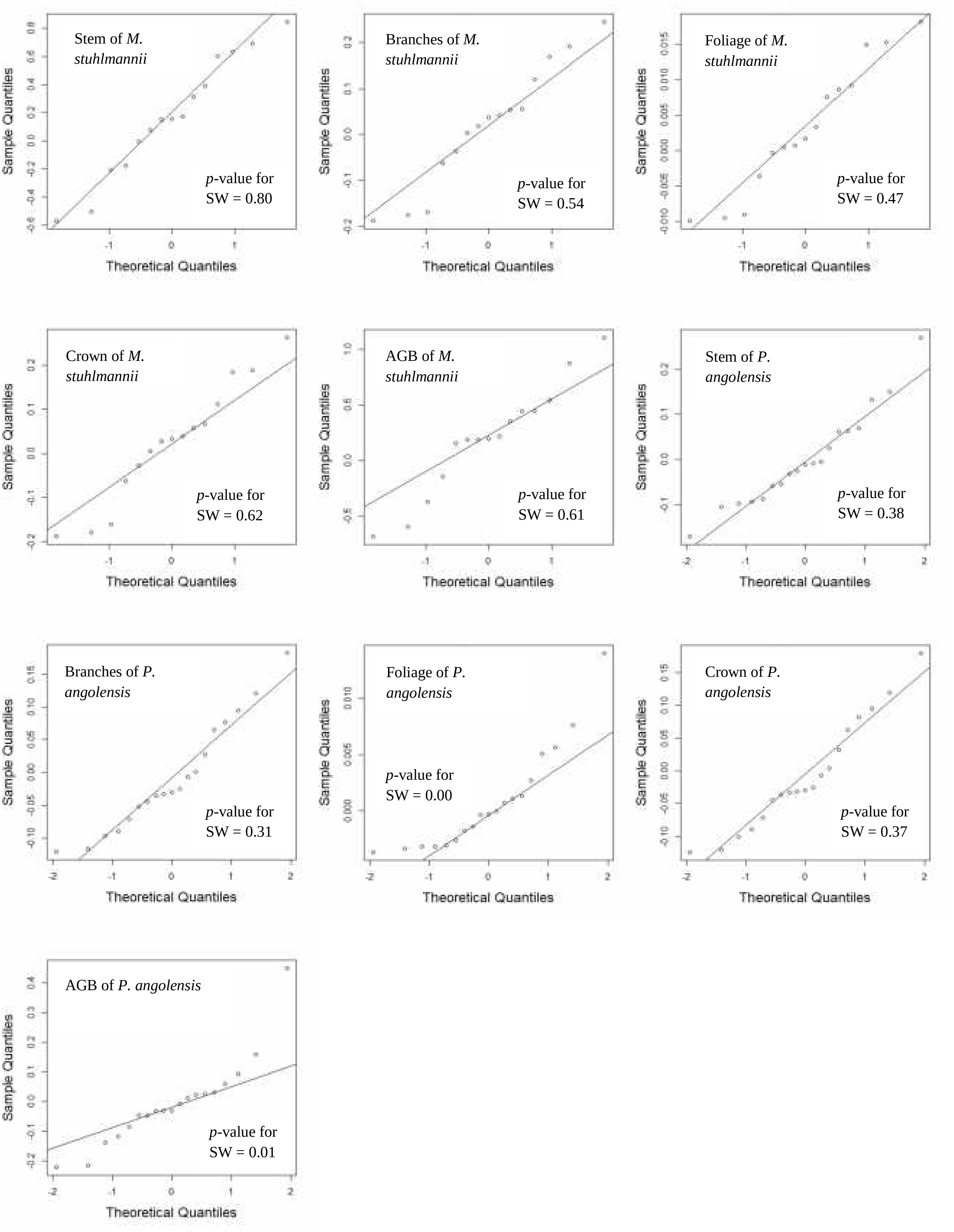

Appendix 4: Residual Quantile-Quantile (Q-Q) plots and Shapiro Wilk (SW) normality test for tree component biomasses of *M. stuhlmannii* and *P. angolensis.*
